# Performance in Solar Orientation Determination for Regular Pyramid Sun Sensors

**DOI:** 10.3390/s19061424

**Published:** 2019-03-22

**Authors:** Jiang Wang, Yongchao Zhang, Yin Zhang, Yulin Huang, Jianyu Yang, Yuming Du

**Affiliations:** 1 School of Communication and Information Engineering, University of Electronic Science and Technology of China, Chengdu 610054, Sichuan, China; rivers2000@163.com (J.W.); yulinhuang@uestc.edu.cn (Y.H.); jyyang@uestc.edu.cn (J.Y.); 2 School of Electronic Engineering, Chengdu University of Information Technology, Chengdu 610225, Sichuan, China; dym@cuit.edu.cn

**Keywords:** sun sensors, solar orientation, regular pyramid arrays

## Abstract

Non-planar sun sensors can determine solar orientation by existing photodiodes or by reusing solar panels, without increasing the size and mass of spacecraft. However, a limiting factor for the improvement of the accuracy of orientation lies in the lack of a detailed performance assessment on interference suppression. In this paper, a new method that determines solar orientation in the frequency domain is developed for regular pyramid sun sensors, which are formed by regular pyramid arrays. Furthermore, two formulations are established to evaluate the errors of the solar azimuth and elevation angle in solar orientation determination based on the newly proposed frequency-domain method. With these formulations of performance evaluation, we discover the mathematical relationship between the interference spectrum, array geometry, solar irradiance, solar azimuth or elevation angle, and the error in solar orientation determination for the first time. This reveals that the internal interference from the detection system can be completely suppressed in solar orientation determination, and the constant interference can be eliminated in the estimation of solar azimuth angle. Simulation and field experiments validated the effectiveness of the new orientation method, error formulations and performance of each interference source.

## 1. Introduction

**Figure 1:**
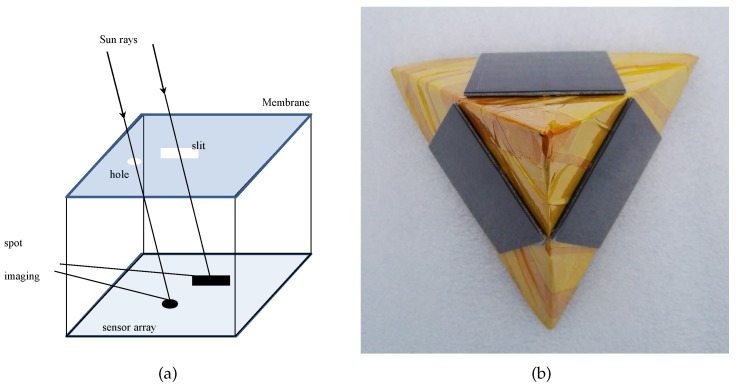
The structure of sun sensors. (**a**) The planar array. (**b**) The triangular pyramidal array.

Sun sensors, which can be formed by planar or non-planar sensor arrays, have a wide range of applications, such as the attitude control of satellites [1,2], assisted positioning for planetary rovers [3], ground-based navigation systems [4], and the efficiency improvement of solar power plants [5]. In the aerospace field, sun sensors formed by planar sensor arrays are primarily used to obtain high-accuracy solar orientation. These sun sensors determine solar orientation by the optical imaging position of the Sun on the planar array through a small masking hole or slit. Examples include complementary metal-oxide semiconductors (CMOSs) [6], charge-coupled-devices (CCDs) [7], and micro-electro-mechanical systems (MEMS) [8]. According to the structure of these sun sensors shown by Figure 1a, the heights of the masking hole or slit, above the planar array, are larger than zero, which limits their detectable field of view (FOV) to less than $180^{{}^{\circ}}$. Thus, at least three such sun sensors are required to determine the Sun’s position for full FOV applications, which generates an extra load for small aerospace equipment, such as nanosatellites in terms of limited size and weight.

Non-planar sun sensors determine solar orientation by the irradiance of the Sun passing through the planes of sensors, such as photodiodes, that are mounted on a non-planar array, such as the triangular pyramidal array shown in Figure 1b. These sun sensors are usually constructed by photodiodes mounted on different surfaces of spacecraft [1,2] or sometimes by the direct reuse of the spacecraft solar panels [9], which hardly generates an extra load for small aerospace equipment. However, due to its susceptibility to interference, these sun sensors are primarily used in spacecraft that do not require high-accuracy solar orientation determination or for the ground tracking of the Sun [5].

The error in solar orientation determination for non-planar sun sensors comes from two interference sources: (1) internal interference which may originate from measurement devices in sun sensors, including misalignment, an undesired scale factor of sensors, such as photodiodes, and an imperfect surrounding circuitry; and (2) external interference, which originates from the surrounding environment, including scattered and reflected sunlight, and interfering light sources, such as an infrared radiation source [10,11,12,13,14]. Modeling all interferences in solar orientation determination as zero-mean Gaussian noise [11,12,13,14], calibrations for the misalignment and undesired scale factor of photodiodes [11,12] and optimizations for the design of the non-planar sensor arrays have been developed [13,14]. These works show that higher accuracy of orientation can be achieved by increasing the number of illuminated photodiodes on the non-planar sensor array [4,13] and that the error of orientation varies with direction of the Sun [12,14]. As a result, using uniform and symmetric configurations as well as more photodiodes to design the non-planar sun sensor array is suggested for improving the accuracy of orientation [14]. However, these works only provide ways to suppress the zero-mean Gaussian noise.

The internal or external interferences of non-planar sun sensors are hardly modeled as zero-mean Gaussian noise. As the angles between the Sun’s direction and the normal direction of each photodiode remain unknown, the irradiance of the Sun passing through each photodiode is unknown. Thus, the interference from the undesired scale factor of photodiodes is not a zero-mean error, and the internal interference is therefore not a zero-mean error either. The internal interference fundamentally limits the non-planar sun sensors, but, to date, there is no good way to eliminate it. Generally, the external interference could include some constant interference, which may come from scattered sunlight in the atmosphere or sensor planes, illuminated by reflected sunlight, such as the earth albedo in space. These constant external interferences from the surrounding environment, especially the atmosphere, could be the major source of error in solar orientation determination, but the study for suppressing it is still lacking.

In order to address the above problems, this study first proposes a new solar orientation method in the frequency domain, based on regular pyramid sensor arrays. Secondly, we establish two mathematical formulations to depict the errors in azimuth and elevation angles in solar orientation determination. Thirdly, we model interference sources of non-planar sun sensors and then assess the performance of each interference source. Finally, we test the orientation method and the performance of interference suppression through simulations and field experiments.

The rest of this paper is organized as follows. In Section 2, the method for determining the solar orientation in the frequency domain is introduced by using regular pyramid sensor arrays. The error formulations of solar azimuth and elevation angles are established in Section 3. The model of the source interference of non-planar sun sensors is established, and the performance achieved by suppressing each interference source is assessed in Section 4. Section 5 provides a verification of the orientation method, error formulations, and the performance achieved by suppressing each interference source by simulations and field experiments. Section 6 concludes this study with a summary and discussions.

## 2. Frequency-Domain Solar Orientation Method

The Sun is far enough from the observation point that the wave front reaching the observation point can be assumed to be parallel. Thus, we define the sun vector to be pointing towards the Sun from the observation point with a magnitude equaling the solar irradiance. Note that the sun vector in this work is defined as being in the opposite direction of sunlight, while the traditional sun vector [4] is its unit vector.

**Figure 2:**
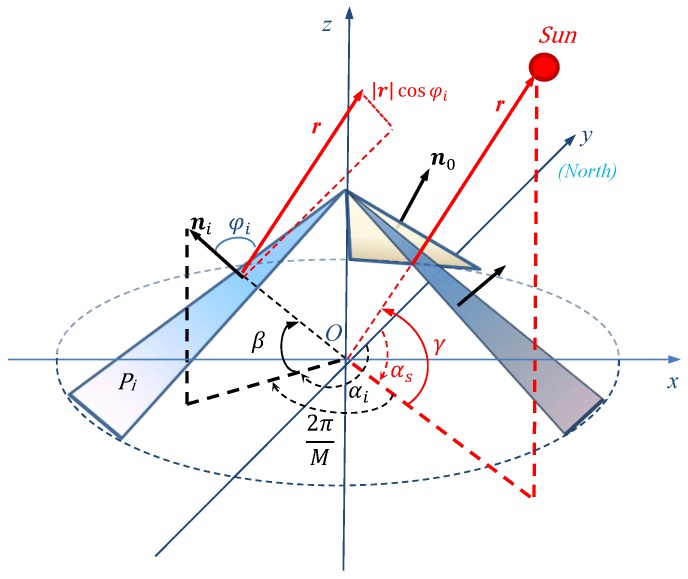
Geometric relationship of the sun vector and the regular pyramid sensor array in an x-y-z Cartesian coordinate system.

The geometric relationship between the sun vector and the regular pyramid sensor array, in an x-y-z Cartesian coordinate system, is shown in Figure 2. The x-y-z Cartesian coordinate system is established using the bottom of a regular pyramid, which is formed by *M* (M≤3) lateral sides as the x-y plane and the center of the bottom as the origin *O*. In the system, the sun vector r has an azimuth angle $\alpha_{s}$ and an elevation angle γ. The illuminated sensor plane $P_{i}$ (where i∈{0,2,…,M−1}) is mounted on a lateral side of the regular pyramid, at an azimuth angle $\alpha_{i}$ and elevation angle β; and its unit normal vector $n_{i}$ aligns with the local vertical. r makes the angle $\varphi_{i}$ with $n_{i}$. The azimuth angle is the angle from true north (if applied on the Earth), noted here again as the positive y direction, which rotates to the east to a projected vector on the x-y plane. The elevation angle is the angle between a vector and x-y plane. The *P* sensor planes are numbered from 0 to *M*-1 in ascending order according to the normal azimuth in these planes.

According to the cosine law for radiation [15], the irradiance passing vertically through the sensor plane is $\left|r\right|\cos\varphi_{i}$, which is equal to the inner product of the sun vector and the unit normal vector of the sensor plane, $r\cdot n_{i}=\left|r\right|\cos\varphi_{i}$. The irradiance that passes through a sensor plane $P_{i}$ can be assumed to be

(1) $$x_{i}=\left|r\right|\cos\varphi_{i}$$

Substituting $r\cdot n_{i}=\left|r\right|\cos\varphi_{i}$ in Equation (1), we can further obtain the following equation for the sensor array

(2) $$x_{i}={n_{i}}^{T}r$$

where $n_{i}={\left(\sin\alpha_{i},\cos\beta ,\cos\alpha_{i},\cos\beta ,\sin\beta \right)}^{T}$ and $r=\left|r\right|{\left(\sin\alpha_{s},\cos\gamma ,\cos\alpha_{s},\cos\gamma ,\sin\gamma \right)}^{T}$ according to the geometric relationships shown in Figure 2.

As the angle between the normal of each sensor plane, mounted on the adjacent lateral sides of the regular pyramid, is the same, $\alpha_{i}=2\pi i/M+\alpha_{0}$, the following equation can then be derived by Equation (2).

(3) $$x_{i}=\left(\left|r\right|\cos\gamma \cos\beta \cos\left(2\pi i/M+\alpha_{0}-\alpha_{s}\right)+\left|r\right|\sin\beta \sin\gamma \right)$$

Let $a=\left|r\right|\cos\gamma \cos\beta$ and $c=\left|r\right|\sin\beta \sin\gamma$, then we have

(4) $$x_{i}=a\cos\left(2\pi i/M+\alpha_{0}-\alpha_{s}\right)+c$$

According to the ascending order of *i*, we arrange $x_{i}$ into a sequence. For convenience in the following discussions, we give the sequence as the orientation sequence, denoted by x(n), which can be expressed as:

(5) $$x\left(n\right)=\left\{\begin{array}{c} a\;\;\;\cos(2\pi n/M+\alpha_{0}-\alpha_{s})+c,0\leq n\leq M-1\\ 0,\;\;otherwise \end{array}\right.$$

Assume that $X(e^{j\omega })$ is the Fourier transform or spectrum of x(n). By the discrete Fourier transform [16], we have

(6) $$X\left(e^{j\omega }\right)=\sum_{n=0}^{M-1}{x(n)e}^{-j\omega n}$$

Since $a\cos\left(2\pi n/M+\alpha_{0}-\alpha_{s}\right)=a\left(e^{j(2\pi n/M+\alpha_{0}-\alpha_{s})}+e^{-j(2\pi n/M+\alpha_{0}-\alpha_{s})}\right)/2$, we can calculate $X(e^{j\omega })$ by Equation (6) as:

(7) $$X\left(e^{j\omega }\right)=\frac{a}{2}\left[e^{j\left(\alpha_{0}-\alpha_{s}\right)}G\left(e^{j\left(\omega -\frac{2\pi }{M}\right)}\right)+e^{-j\left(\alpha_{0}-\alpha_{s}\right)}G\left(e^{j\left(\omega +\frac{2\pi }{M}\right)}\right)\right]+cG\left(e^{j\omega }\right)$$

where $G\left(e^{j\omega }\right)=e^{-j\omega \left(M-1\right)/2}\frac{\sin\left(M\omega /2\right)}{\sin\left(\omega /2\right)}$.

Substituting $a=\left|r\right|\cos\gamma \cos\beta$ and $c=\left|r\right|\sin\beta \sin\gamma$ into Equation (7), we have

(8) $$X\left(e^{j0}\right)=Mc=M\left|r\right|\sin\beta \sin\gamma$$

and

(9) $$X\left(e^{\pm j2\pi /M}\right)=\frac{Ma}{2}e^{\pm j(\alpha_{0}-\alpha_{s})}=\frac{1}{2}M\left|r\right|\cos\gamma \cos\beta e^{\pm j(\alpha_{0}-\alpha_{s})}$$

where $X(e^{j0})$ is the spectrum of χ(n) at the zero angular frequency, and $X(e^{\pm j2\pi /M})$ is the spectrum component of x(n) at fundamental angular frequencies 2π/M and −2π/M. Note that the two fundamental angular frequencies vary with the number of sensor planes mounted on the regular pyramid.

According to Equation (9), the solar azimuth angle can be obtained from the phases of the spectrum component of x(n) at a fundamental angular frequency of 2π/M or −2π/M, then we have

(10) $$\alpha_{s}=\alpha_{0}\mp X\left(e^{\pm j2\pi /M}\right)$$

Because 0≤γ<π/2, which makes arctan(sinγ/cosγ)=γ, the solar elevation angle can be expressed by

(11) $$\gamma =\arctan\frac{1}{2\tan\beta }\left(\left|X(e^{j0})\right|/\left|X(e^{\pm j2\pi /M})\right|\right)$$

In the Cartesian coordinate system shown in Figure 2, $\alpha_{0}$ and β are known. Therefore, we can conclude from Equations (10) and (11) that the position of the Sun can be determined by the spectrum of the orientation sequence at the zero angular frequency and one of the fundamental angular frequencies based on a regular pyramid sensor array.

For a sensor array using similar sensors, such as photodiodes, the scale factor of sensors may be reasonably assumed to be a constant $\eta \left(\eta >0\right)$. Then, the measurement value of solar irradiance is $\eta \left|r\right|$. According to Equations (8), (9), and (11), the solar azimuth and elevation angles are independent of η. Therefore, the position of the Sun can be determined by measuring the irradiances that pass through sensor planes on the regular pyramid sensor array as well.

## 3. Error Formulation for the Solar Orientation

In applications, the measured irradiance is always influenced by interference [10,11,12]. For the irradiance that passes that passes through a sensor plane $P_{i}$, the output measurement value ${\tilde{s}}_{i}$ can be expressed as:

(12) $${\tilde{s}}_{i}=\eta x_{i}+e_{i}$$

where η is the ideal value of the scale factor of sensors, and $e_{i}$ is the measurement error. According to the ascending order of *i*, we arrange ${\tilde{s}}_{i}$ into a sequence, denoted by $\tilde{s}\left(n\right)$. Then we have:

(13) $$\tilde{s}\left(n\right)=s\left(n\right)+e\left(n\right)$$

where $s\left(n\right)=\eta x_{n}$, 0≤n≤M−1, and $e\left(n\right)=e_{n}$, 0≤n≤M−1.

Let $\tilde{s}$ $\left(e^{j\omega }\right)$ be the spectrum of $\tilde{s}$(n), and $E(e^{j\omega })$ be the spectrum of e(n). According to the linearity of the discrete Fourier transform, the spectrum of $\tilde{s}$(n) can be obtained from Equation (13) as follows

(14) $$\tilde{S}\left(e^{j\omega }\right)=S\left(e^{j\omega }\right)+E\left(e^{j\omega }\right)$$

Substituting $S(e^{j0})$ into Equation (14), the spectrum component of $\tilde{s}$(n) at the zero angular frequency is given by

(15) $$\tilde{S}\left(e^{j0}\right)=M\eta \left|r\right|\sin\beta \sin\gamma +E\left(e^{j0}\right)$$

Similarly, substituting $S(e^{\pm j2\pi /M})$ into Equation (14), the spectrum components of $\tilde{s}\left(n\right)$ at fundamental angular frequencies ±2π/M are calculated as

(16) $$\tilde{S}\left(e^{\pm j2\pi /M}\right)=1/2M\eta \left|r\right|\cos\gamma \cos\beta e^{j(\alpha_{0}-\alpha_{s})}+E\left(e^{j2\pi /M}\right)$$

### 3.1. Error Formulation for the Solar Azimuth Angle

According to the formulation in Equation (10) of the solar azimuth angle, the error of the solar azimuth angle in solar orientation determination is the absolution value of the difference between the phases of $S(e^{j2\pi /M})$ and $\tilde{S}$ $\left(e^{j2\pi /M}\right)$. In a complex plane, the magnitude of complex number $\tilde{S}$ $\left(e^{j2\pi /M}\right)$, $S(e^{j2\pi /M})$, or $E(e^{j2\pi /M})$ is equal to its amplitude, while the argument is equal to its phase. Thus, we define the absolute value of the difference between the arguments of $S(e^{j2\pi /M})$ and $\tilde{S}$ $\left(e^{j2\pi /M}\right)$ to describe the error in the solar azimuth angle. This is denoted as θ.

**Figure 3:**
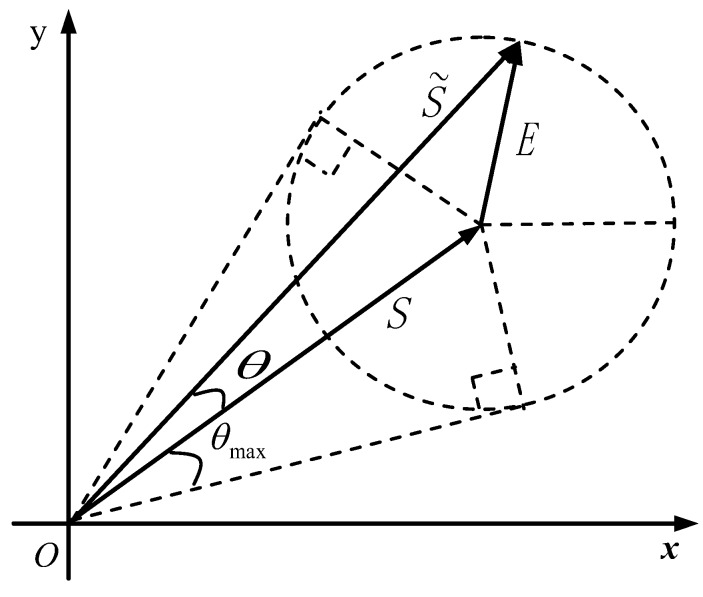
Geometric relations of $\tilde{S}$, S, and E on the complex plane.

Usually, $\left|E(e^{j2\pi /M})\right|$ is less than $\left|S(e^{j2\pi /M})\right|$ because of the energy of s(n) is much greater than e(n) in practical applications. Thus, we may assume that $\left|E(e^{j2\pi /M})\right|<\left|S(e^{j2\pi /M})\right|$ is satisfied in the following discussions. From Equation (14), the geometric relations between $\tilde{S}$ $\left(e^{j2\pi /M}\right)$, $S(e^{j2\pi /M})$, and $E(e^{j2\pi /M})$ in a complex plane can be shown as in Figure 3, where $\tilde{S}$ $\left(e^{j2\pi /M}\right)$, $S(e^{j2\pi /M})$, and $E(e^{j2\pi /M})$ are denoted by $\tilde{S}$, S, and E, respectively. The value of θ varies with E. When E is perpendicular to $\tilde{S}$, θ reaches its maximum value (labeled as $\theta_{\max}$) for the same $\left|E\right|$; when E is of the same or opposite direction of S, θ is zero. According to the geometric relationships shown in Figure 3, we have:

(17) $$\theta \leq \theta_{\max}=\arcsin\frac{\left|E(e^{j2\pi /M})\right|}{\left|S(e^{j2\pi /M})\right|}=\arcsin\frac{\left|E(e^{\pm j2\pi /M})\right|}{\left|S(e^{\pm j2\pi /M})\right|}$$

For convenience, we denote the supremum (i.e., the least upper bound) of error in the solar azimuth angle in Equation (17) as $\theta_{\sup}$ and express it as

(18) $$\theta_{\sup}=\arcsin\frac{\left|E(e^{\pm j2\pi /M})\right|}{\left|S(e^{\pm j2\pi /M})\right|}$$

Substituting $\left|S(e^{\pm j2\pi /M})\right|$, which is expressed in Equation (9), into Equation (18), we have:

(19) $$\theta_{\sup}=\arcsin\frac{2\left|E(e^{\pm j2\pi /M})\right|/M}{\eta \left|r\right|\cos\gamma \cos\beta }$$

where β is the angle between the normal of the sensor planes and the x-y plane; again, *M* is the number of sensor planes, which is determined by the special configuration of the sensor array; $\left|E(e^{j2\pi /M})\right|$ is the magnitude of the spectrum component of the interference sequence at any fundamental angular frequency; $\eta \left|r\right|$ relates to the measurement value of the irradiance; and γ is solar elevation angle. Equation (19) indicates that the error in the solar azimuth angle comes from the spectrum component of an interference sequence at fundamental angular frequencies, and is related to the irradiance measurement, solar elevation angle, and the special configuration of the sensor array. Since γ or β is greater than 0, cosβ or cosγ is smaller than 1. Thus, $\theta_{\sup}$ is amplified by γ or β. As the result, the larger γ or β are, the greater the amplification of the supremum for the error in the solar azimuth angle.

#### Error Formulation for the Solar Elevation Angle

Similarly, according to the formulation in Equation (11) for the solar elevation angle, the estimation $\tilde{\gamma }$ can be obtained by substituting $\tilde{S}$ $\left(e^{j0}\right)$ and $\tilde{S}$ $\left(e^{\pm j2\pi /M}\right)$, which are expressed in Equations (15) and (16), respectively, into Equation (11) and can be expressed as:

(20) $$\tilde{\gamma }=\arctan\frac{\left|\sin\gamma +\frac{E(e^{j0})/M}{\eta \left|r\right|\sin\beta }\right|}{\left|\cos\gamma +\frac{2e^{-j(\alpha_{0}\mp \alpha_{s})}E\left(2e^{\pm j2\pi /M}\right)/M}{\eta \left|r\right|\cos\beta }\right|}$$

where $E(e^{j0})$ and $E(e^{\pm j2\pi /M})$ are the spectrum components of the interference sequence at angular frequencies of 0 and ±2π/M, respectively; and α is the solar azimuth angle. The error in the solar elevation angle, i.e., the difference between $\tilde{\gamma }$ and γ, can be expressed as

(21) $$\zeta =\left|\arctan\frac{\left|\sin\gamma +\frac{E(e^{j0})/M}{\eta \left|r\right|\sin\beta }\right|}{\left|\cos\gamma +\frac{2e^{-j(\alpha_{0}\mp \alpha_{s})}E\left(2e^{\pm j2\pi /M}\right)/M}{\eta \left|r\right|\cos\beta }\right|}-\gamma \right|$$

Equation (21) indicates that the error in the solar elevation angle comes from the spectrum components of the interference sequence, at angular frequencies of 0 and ±2π/M, and is related to the irradiance measurement, solar azimuth angle, and the special configuration of the sensor array. Because β∈(0,π/2), we have 0<sinβ<1 and 0<cosβ<1, which will amplify ζ as $E(e^{j0})$ = 0 or $E(e^{j2\pi /M})$ = 0. When β increases, the error in the solar elevation angle decreases in the case of $E(e^{j2\pi /M})$ = 0 but increases with the same $\alpha_{s}$ in the case of $E(e^{j0})$ = 0. We note that when $E(e^{j0})\neq 0$ and $E(e^{j2\pi /M})\neq 0$, the error in the solar elevation angle could be 0, because $\left|\sin\gamma +\frac{E(e^{j0})/M}{\eta \left|r\right|\sin\beta }\right|/\left|\cos\gamma +\frac{2e^{-j(\alpha_{0}\mp \alpha_{s})}E\left(2e^{\pm j2\pi /M}\right)/M}{\eta \left|r\right|\cos\beta }\right|$ could be equal to $\left|\sin\gamma \right|/\left|\cos\gamma \right|$.

## 4. Interference Suppression Performance

As mentioned earlier, the error in the orientation of non-planar sun sensors can come from internal and external interference. In the following section, we discuss the performance of regular pyramid sun sensors and the suppression of internal and external interference. As the Sun and its reflected light usually reach the observation point via different paths, we can assume that the reflected sunlight is an interfering light source in the surrounding environment. In the atmosphere, scattering sunlight consists of much reflected sunlight shining in different directions. Similarly, we can assume that it has many interfering light sources that shine in different directions. Thus, we will discuss the suppression of the interfering light source for external interference.

### 4.1. Solar Orientation Determination Interference Modeling

The internal interference varies with the product of the irradiance, measured on sensor planes, and the disturbance from the misalignment and undesired scale factor of sensors and an imperfect surrounding circuitry. On the contrary, the external interference only varies with the interfering light source in the surrounding environment. Thus, the interference sequence can be expressed as

(22) $$e\left(n\right)=f\left(n\right)\left[s\left(n\right)+e_{0}\left(n\right)\right]+e_{0}\left(n\right)$$

where f(n) is the sequence formed by errors of misalignments and the scale factors of sensors, and the output error of the surrounding circuitry; and $e_{0}\left(n\right)$ is a sequence composed of the true measurement of the irradiance of interfering light sources passing through the sensor planes. Usually, elements of f(n) are far less than 1. For example, the value of a solar plane is less than ±0.05. Thus, interference sequence can be approximately expressed as

(23) $$e\left(n\right)=f\left(n\right)s\left(n\right)+e_{0}\left(n\right)$$

Then, the Fourier transform of e(n) is derived using the convolution property of the discrete Fourier transform as

(24) $$E\left(e^{j\omega }\right)=\frac{1}{M}\sum_{k=-1}^{1}S\left(e^{j\frac{2\pi k}{M}}\right)F\left(e^{j(\omega +\frac{2\pi k}{M})}\right)+E_{0}\left(e^{j\omega }\right)$$

where *M* is again the number of sensor planes on the sensor array, $S(e^{j\frac{2\pi k}{M}})$ is the spectrum component of s(n) at an angular frequency of $\frac{2\pi k}{M}$; $F(e^{j(\omega +\frac{2\pi k}{M})})$ is the spectrum component of s(n) at an angular frequency of $\omega +\frac{2\pi k}{M}$; $E_{0}\left(e^{j\omega }\right)$ is the Fourier transform of $e_{0}\left(n\right)$. For convenience, we denote $E_{in}\left(e^{j\omega }\right)$ as $\frac{1}{M}\sum_{k=-1}^{1}S\left(e^{j\frac{2\pi k}{M}}\right)F\left(e^{j(\omega +\frac{2\pi k}{M})}\right)$, which is the spectrum generated by internal interference, i.e., the Fourier transform of f(n)s(n). Then, $E(e^{j\omega })$ can be simplified as:

(25) $$E\left(e^{j\omega }\right)=E_{in}\left(e^{j\omega }\right)+E_{0}\left(e^{j\omega }\right)$$

### 4.2. Performance of Internal Interference Suppression

The disturbance from the misalignment and undesired scale factor of sensors and an imperfect surrounding circuitry on each sensor plane is usually random and independent. Thus, we may model f(n) as zero-mean white Gaussian noise and take its power spectrum $\left({\left|F(e^{j\omega })\right|}^{2}/M\right)$ as a constant, which makes $\left|F(e^{j\omega })\right|/\sqrt{M}$ into a constant as well. Since $S\left(e^{j\frac{2\pi k}{M}}\right)/M\left(k=0\;or\;k=\pm 1\right)$ is a constant, which is obtained from Equation (9), and $\left|E_{in}\left(e^{j\omega }\right)\right|/\sqrt{M}$ which can be expressed as $\left|\sum_{k=-1}^{1}\frac{S(e^{j\frac{2\pi k}{M}})}{M}\frac{F(e^{j(\omega +\frac{2\pi k}{M})})}{\sqrt{M}}\right|$, is a constant as well, we can deduce that $\left|E_{in}\left(e^{j\omega }\right)/M\right|$ is inversely proportional to $\sqrt{M}$ and satisfies

(26) $$\lim_{M\to \infty }\left|E_{in}\left(e^{j\omega }\right)/M\right|=0$$

From Equation (26), we can conclude that the spectrum amplitude of the sequence generated by internal interference is inversely proportional to the square root of the number of sensor planes on the regular pyramid. Therefore, the internal interference can always be eliminated by increasing the sensor planes.

### 4.3. Performance of External Interference Suppression

Considering that the Sun is a light source, we may regard the Sun shown in Figure 2 as an light source to derive the expression of $e_{0}\left(n\right)$. To distinguish between the interfering light source and the Sun, we assume that the interfering light source has an azimuth angle $a_{e}$, elevation angle $\gamma_{e}$, and a vector $r_{e}$ pointing towards the interfering light source from the observation point, with a magnitude of its irradiance like the sun vector. According to Figure 2, sensor planes on the regular pyramid are illuminated in whole or in part when $\gamma_{e}$ takes on different values. In the following, we discuss the suppression of the interfering light source in two scenarios: sensor planes illuminated in whole and in part.

Using the same derivation of x(n) in Section 2, from Equation (6) in the case of sensor planes on the regular pyramid illuminated in whole, $e_{0}\left(n\right)$ can expressed as follows:

(27) $$e_{0}(n)=\left\{\begin{array}{c} a_{e}\;\;\cos\left(2\pi n/M+\alpha_{0}-\alpha_{s}\right)+c_{e},0\leq n\leq M-1\\ 0,\;\;otherwise \end{array}\right.$$

where $a_{e}=\eta \left|r_{e}\right|\cos\gamma_{e}\cos\beta$, $c_{e}=\eta \left|r_{e}\right|\sin\beta \sin\gamma_{e}$, and β is the angle between the normal of any sensor plane on the regular pyramid and the x-y plane. Similarly, we can derive that $E_{0}\left(e^{j0}\right)/M=\eta \left|r_{e}\right|\sin\beta \sin\gamma_{e}$ and $E_{0}\left(\pm j2\pi /M\right)/M=\frac{1}{2}\eta \left|r_{e}\right|\cos\gamma_{e}\cos\beta e^{\pm j\alpha_{0}-\alpha_{s}}$ by Equations (8) and (9), and then conclude that $E_{0}\left(e^{j0}\right)/M$ and $E_{0}\left(\pm j2\pi /M\right)/M$ are independent of *M*, and the errors for the solar azimuth and elevation angles are independent of β, according to Equations (19) and (21). In particular, when $\gamma_{e}=90^{{}^{\circ}}$, $e_{0}\left(n\right)$ equals the constant $c_{e}$, $E_{0}\left(\pm j2\pi /M\right)$ equals 0. Thus we can conclude that the error in the solar azimuth angle is independent of an interfering light source, with $\gamma_{e}=90^{{}^{\circ}}$ by Equation (19).

The second scenario, with regard to an interfering light source, is the case of sensor planes illuminated in part. Let the number of illuminated sensor planes be *N*, where N<M. Since illuminated sensor planes are adjacent to each other, and the irradiance on the sensor plane that is not illuminated by the interfering light source is 0, according to Figure 2, we have:

(28) $$e_{0}\left(n\right)=\left\{\begin{array}{c} a_{e}\;\;\cos\left(2\pi n/M+\alpha_{0}-\alpha_{s}\right)+c_{e},N_{1}\leq n\leq N_{1}+N-1\\ 0,\;\;otherwise \end{array}\right.$$

where the sensor planes numbered from $N_{1}$ to $N_{1}+N-1$ are illuminated. Similarly, we have

(29) $$e_{0}\left(n\right)=\left\{\begin{array}{c} 0,\;\;N_{2}\leq n\leq N_{2}+M-N-1\\ a_{e}\;\;\cos\left(2\pi n/M+\alpha_{0}-\alpha_{s}\right)+c_{e},\;\;otherwise \end{array}\right.$$

where the sensor planes numbered from $N_{2}$ to $N_{2}+M-N-1$ are not illuminated. According to the discrete Fourier transform, $E_{0}\left(e^{j\omega }\right)$ can be expressed as $\sum_{n=N_{1}}^{N_{1}+N-1}e_{0}\left(n\right)e^{-j\omega n}$ or $\sum_{n=0}^{M-1}e_{0}\left(n\right)e^{-j\omega n}-\sum_{n=N_{2}}^{N_{2}+M-N-1}e_{0}\left(n\right)e^{-j\omega n}$ by Equation (28) or (29). Using calculations similar to those employed in obtaining Equation (8) and (9), we can write:

(30) $$E_{0}\left(e^{j0}\right)=\frac{a_{e}\sin\frac{\pi N}{M}}{\sin\frac{\pi }{M}}\cos\left(\frac{\pi (2N_{1}+N-1)}{M}+a_{0}-a_{e}\right)+Nc_{e}$$

(31) $$E_{0}\left(e^{\pm j\frac{2\pi }{M}}\right)=\frac{a_{e}}{2}\left(Ne^{\pm j(a_{0}-a_{e})}\pm e^{\mp j(\frac{2\pi (2N_{1}+N-1)}{M}+a_{0}-a_{e})}\frac{\sin\frac{2\pi N}{M}}{\sin\frac{2\pi }{M}}\right)\pm c_{e}e^{\mp j\frac{2\pi (2N_{1}+N-1)}{M}}\frac{\sin\frac{\pi N}{M}}{\sin\frac{\pi }{M}}$$

where the sensor planes, numbered from $N_{1}$ to $N_{1}+N-1$ are illuminated, and

(32) $$E_{0}\left(e^{j0}\right)=\frac{a_{e}\sin\frac{\pi N}{M}}{\sin\frac{\pi }{M}}\cos\left(\frac{\pi (2N_{2}+N-1)}{M}+a_{0}-a_{e}\right)+Nc_{e}$$

(33) $$E_{0}\left(e^{\pm j\frac{2\pi }{M}}\right)=\frac{a_{e}}{2}\left(Ne^{\pm j(a_{0}-a_{e})}\pm e^{\mp j(\frac{2\pi (2N_{2}+N-1)}{M}+a_{0}-a_{e})}\frac{\sin\frac{2\pi N}{M}}{\sin\frac{2\pi }{M}}\right)\pm c_{e}e^{\mp j\frac{2\pi (2N_{2}+N-1)}{M}}\frac{\sin\frac{\pi N}{M}}{\sin\frac{\pi }{M}}$$

where the sensor planes, numbered from $N_{2}$ to $N_{2}+M-N-1$ are not illuminated. According to $\lim_{x\to 0}\sin\left(x\right)=x$, we have $\lim_{M\to \infty }\frac{1}{\sin\frac{\pi }{M}}=\frac{M}{\pi }$ and $\lim_{M\to \infty }\frac{1}{\sin\frac{2\pi }{M}}=\frac{M}{2\pi }$. Then, due to *N*, $N_{1}$, and $N_{2}$, proportional to *M*, which can be obtained from Figure 2, we can infer that $E_{0}\left(e^{j0}\right)/M$ and $E_{0}\left(\pm j\frac{2\pi }{M}\right)/M$ are independent of *M* for a large *M* from Equations (30), (31), (32) and (33).

In summary, we can conclude that the interfering light source, with a $90^{{}^{\circ}}$ elevation angle, is independent of the error in the solar azimuth angle, but the errors in solar orientation determination caused by any interfering light source, including errors of the solar azimuth and elevation angles, are independent of the number of sensor planes on the regular pyramid.

## 5. Experiment and Analysis

### 5.1. Simulations

In this section, we validate the error formulations for the solar azimuth and elevation angles in solar orientation determination and the performance of interference suppression by comparing the error of orientation in the case of internal or external interference.

According to the discussion in Section 4, the error sequence f(n) of sensors may be modeled as zero-mean white Gaussian noise, while external interference may originate from an interfering light source. Thus, we consider a random sequence, formed by zero-mean white Gaussian noise, with the power set to an arbitrary value of 20 dB of power to simulate f(n), and an interfering light source with the intensity set to an arbitrary value, such as $\left|r_{e}\right|$=10, with three positions used to simulate external interference from the surrounding environment. According to the expression of external sequence $e_{0}\left(n\right)$, derived in Section 4, $e_{0}\left(n\right)$ can be: (1) a constant sequence, which is generated by an interfering light source, with elevation angle $\gamma_{e}=90^{{}^{\circ}}$; (2) a combination of constant sequence and cosine sequence with *n* from 0 to M−1, which is generated by an interfering light source illuminating all the sensor planes, but $\gamma_{e}<90^{{}^{\circ}}$; or (3) a truncation of (2), which is generated by an interfering light source illuminating the sensor planes in part. To simulate the three kinds of sequences of external interference, the configurations of the positions of these interfering light sources are shown in Table 1. Note that position 1 is unique and used to generate sequence (1), while positions 2 and 3 are optional and used to generate to sequences (2) and (3), respectively.

To validate the influence of *M* and β in solar orientation determination, we design four regular pyramid sensor arrays, where any two arrays have the same *M* or β. The configurations of the four arrays are given in Table 2, where we include two arbitrary values for M=16 or 256, two arbitrary values for $\beta =65^{{}^{\circ}}$ or $80^{{}^{\circ}}$, and an arbitrary value for $\alpha_{0}=0$. In order for all sensor planes on the four arrays to be illuminated, the solar azimuth angle $\alpha_{s}$ increases from $0^{{}^{\circ}}$ to $359^{{}^{\circ}}$, and the solar elevation angle γ first increases from $30^{{}^{\circ}}$ to $80^{{}^{\circ}}$ and then decreases to $30^{{}^{\circ}}$. Due to the irradiance of the Sun $\left|r\right|$ and the fact that the scale factor η of similar sensors can be assumed to be constant, we set an arbitrary value for $\left|r\right|$ = 100 and η = 1 during the simulations.

#### Simulation Results

**Figure 4:**
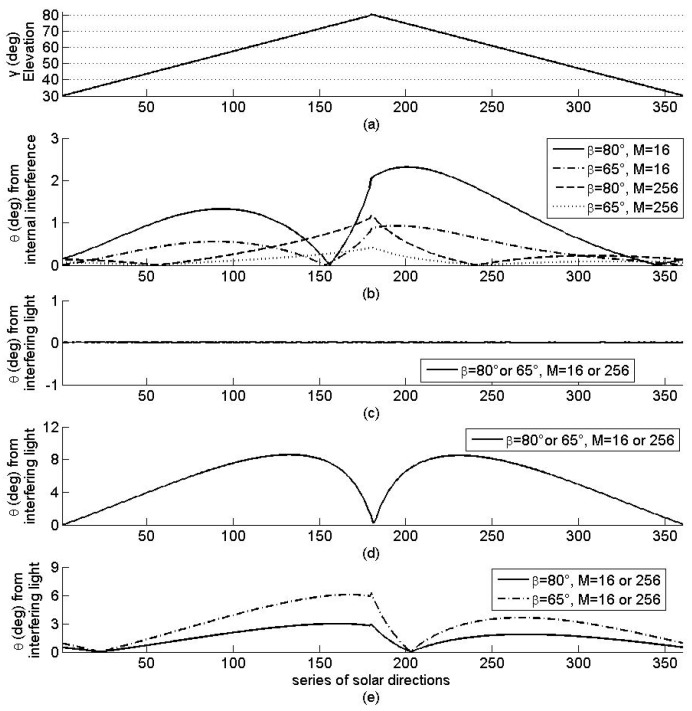
Solar azimuth angle (**a**) and the error θ in the azimuth angle for four arrays with different interference: (**b**) internal interference; (**c**–**e**) external interference caused by interfering light sources at positions 1, 2, and 3 respectively.

The simulation results in the error θ in the solar azimuth angle, as shown in Figure 4 for the four arrays. First, for internal interference, as shown in Figure 4a,b, θ decreases with the increase of *M* (but γ and β remain constant) and increases with the increase of β (but γ and *M* remain constant). Secondly, for the interfering light source at position 1, as shown in Figure 4a,c, θ is 0 and independent of γ, β, and *M*. Thirdly, for the interfering light source at position 2, as shown in Figure 4a,d, θ varies with γ and is independent of β and *M*. Finally, for the interfering light source at position 3, as shown in Figure 4a,e, θ varies with γ and increases with the increase of β, when γ remains constant but is independent of *M*. These results agree with the relationship among θ, γ, β, and *M*.

**Figure 5:**
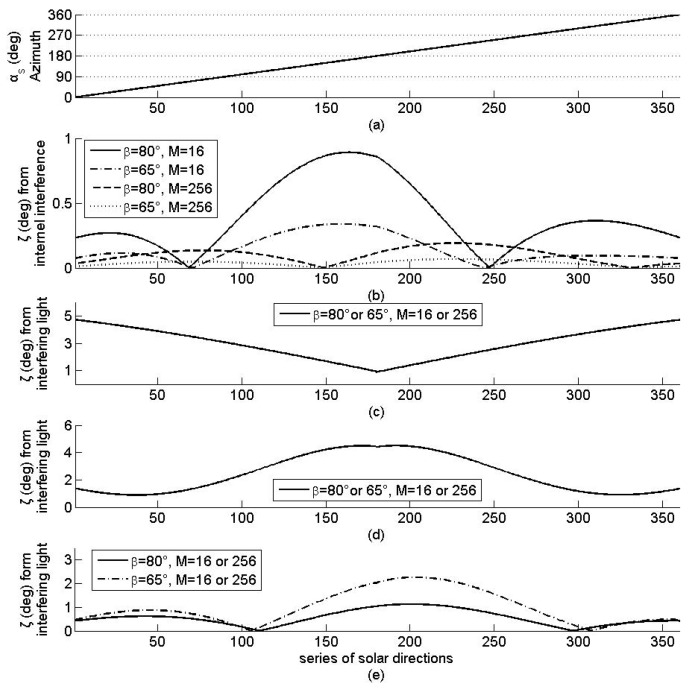
Solar elevation angle (**a**) and the error ζ in the elevation angle for four simulated arrays with different interference: (**b**) internal interference; (**c**–**e**) external interference caused by interfering light sources at positions 1, 2, and 3 respectively.

Similarly, the simulation results concerning the error ζ of solar elevation angle are shown in Figure 5 for the four arrays. Concerning the internal interference, as shown in Figure 5a,b, ζ increases with the increase of β (but α and *M* remain constant), and its oscillation amplitude decreases with the increase of *M* (but β remains constant). As for the interfering light source at positions 1 and 2, as shown in Figure 5a,c,d, ζ varies with $\alpha_{s}$ and is independent of β and *M*. Unlike the error of the solar azimuth angle, ζ is greater than 0 with the interfering light source at position 1. As for the interfering light source at position 3, as shown in Figure 5a,e, ζ varies with β and decreases with the increase of β when $\alpha_{s}$ keeps constant but is independent of *M*. These results validate the relationship among ζ, $\alpha_{s}$, β, and *M*.

From the discussions on Figure 4 and Figure 5, we can conclude that, the error in solar orientation determination is independent of *M*, for any interfering light source, and β for the interfering light source that illuminates all the sensor planes on the sensor array but decreases with the increase of *M* for the internal interference and with the increase of *M* for all interference. The error in the solar azimuth angle is independent of constant interference. These results validate the assertion related to the interference suppression of regular pyramid sun sensors.

### 5.2. Field Experiments for Solar Orientation Determination

In this section, we investigate the performance of the solar orientation method frequency domain and the traditional orientation method, based on the method of using the least squares [4], by comparing the errors in the solar azimuth and elevation angle. In addition, the method of increasing the number of sensor planes on the regular pyramid to optimize the accuracy of orientation is validated using field experiments as well.

**Figure 6:**
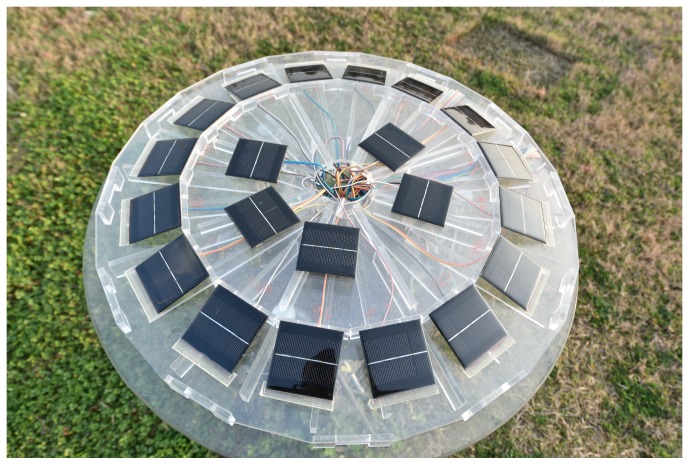
Regular 16-pyramid sun sensors.

Our sensor array is designed such that 16 solar panels are mounted on the lateral surfaces of a regular 16-pyramid (Figure 6), i.e., M=16. From the regular pyramid, we have 7 arrays to determine the solar orientation by the frequency domain method. Their configurations are given in Table 3. To increase the detectable FOV, the angle between the lateral and bottom faces of the pyramid is designed to be $26.4^{{}^{\circ}}$, i.e., $\beta =63.6^{{}^{\circ}}$. The solar panels used are monocrystalline silicon batteries with a voltage of 5V open-circuit and short-circuit current of 160 mA. With the same irradiance, the output error of the solar panels is less than ±5%. The assembly error of various solar panels is less than $\pm 1^{{}^{\circ}}$. Since the short-circuit current outputs of the solar panels vary approximately linearly with the illumination intensity, the solar irradiance on various surfaces of the pyramid is measured by the short-circuit current outputs of the solar panels.

To calculate the position of the Sun, a Cartesian coordinate system is set with the center of the pyramid base as the origin, the ground surface as the x-y coordinate plane, the y-axis pointing toward true north, and the x-axis toward east. In the system, the true azimuth angle $\alpha_{s}$ and elevation angle γ of the Sun are calculated by astronomical formulas, which use the local latitude, solar hour, and solar declination angle [15,17].

**Figure 7:**
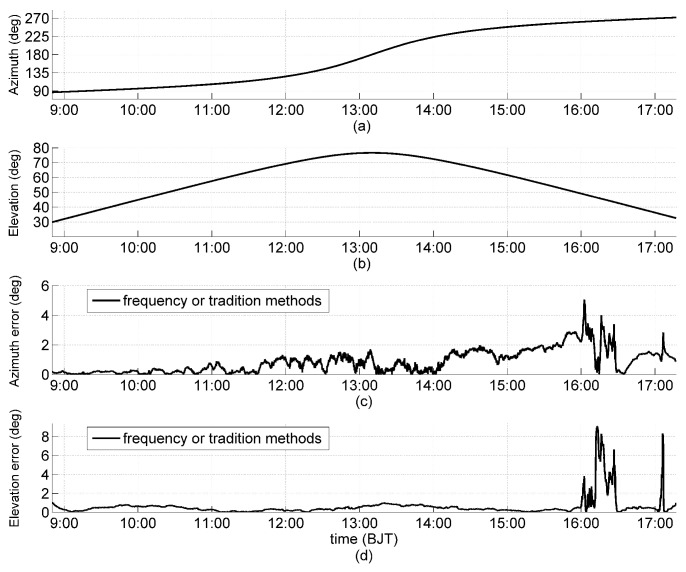
Solar azimuth angle (**a**); elevation angle (**b**); solar experimental orientation error for Array 7 (M=16) comparing the method of frequency analysis to the traditional method, including the errors of the solar azimuth angle (**c**); and the elevation angle (**d**). Data points are at every 10 seconds. Cloud impacts are shown from 1545–1630 and at around 1707 BJT.

The field experiment was conducted in the suburb of Chengdu, China (longitude $130^{{}^{\circ}}59^{\prime }$, latitude $30^{{}^{\circ}}35^{\prime }$). The measurement platform was installed at the meteorological observation site. The measurement was carried out once every second on 15 August 2015, with sunny weather in the morning, thin clouds around the Sun after 1525 BJT (Beijing Time), and thin clouds covering the Sun from 1545–1630 BJT and at around 1707 BJT. All solar panels were illuminated by sunlight within the solar azimuth angle, which ranged from $86.8^{{}^{\circ}}$ to $271.7^{{}^{\circ}}$ during the period of observation (Figure 7a), while the solar elevation angle ranged from $29.9^{{}^{\circ}}$ to $76.7^{{}^{\circ}}$ and then returned to $32.6^{{}^{\circ}}$ (Figure 7b).

The solar orientation was determined every 10 seconds for Array 7 (M=16) by our frequency domain orientation method and the traditional method. The errors of the solar azimuth and elevation angles are shown in Figure 7c,d, respectively. During the observation, the two methods had the same errors of the solar azimuth and elevation angles, which indicates that our method has the same performance as the traditional method in relation to solar orientation determination. Due to constant external interference, from the surrounding environment, on each sensor plane (with a clear sky), the errors of the solar azimuth angle increased from $0.1^{{}^{\circ}}$ to $1.5^{{}^{\circ}}$, with solar elevation angle increasing from $29.9^{{}^{\circ}}$ to $76.7^{{}^{\circ}}$. The result is consistent with the error formulation Equation (20) and the simulation results.

**Figure 8:**
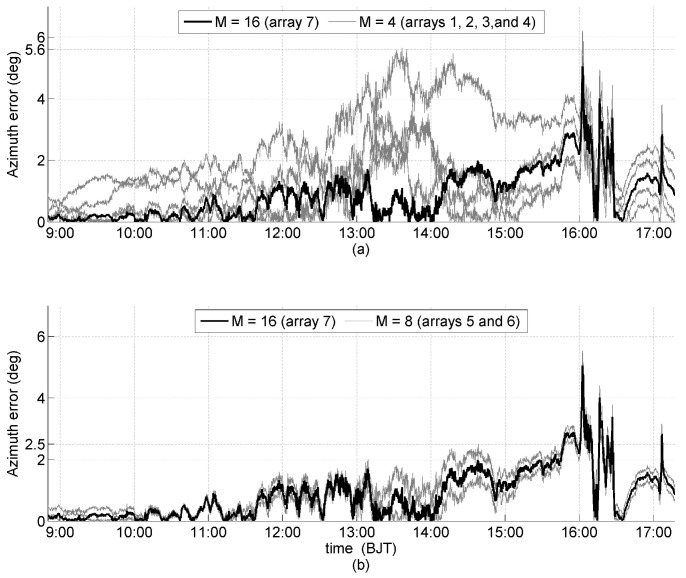
Experimental errors of the solar azimuth angle, with a thick black line generated by Array 7 (M=16), and thin gray lines generated by: (**a**) Arrays 1, 2, 3, and 4 (M=4); and (**b**) Arrays 5 and 6 (M=8).

**Figure 9:**
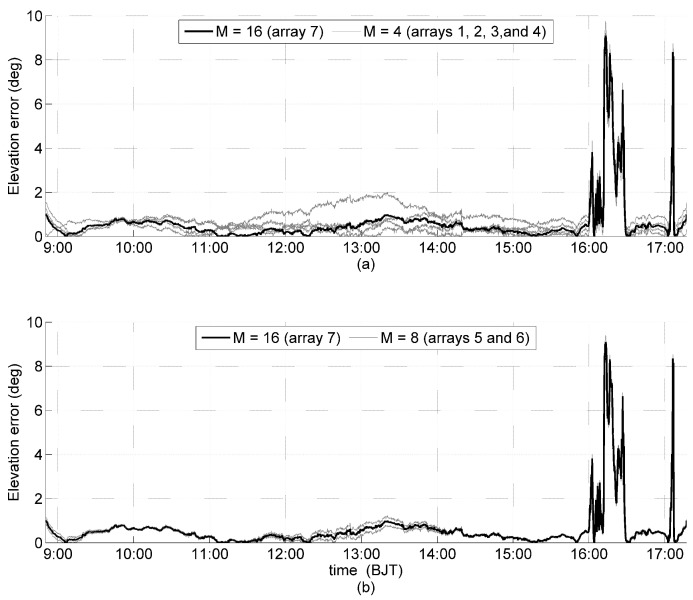
Experimental errors of the solar elevation angle, with a thick black line generated by Array (M=16), and thin gray lines generated by: (**a**) Arrays 1, 2, 3, and 4 (M=4); and (**b**) Arrays 5 and 6 (M=8).

Similarly, the solar orientation was determined every 10 seconds for all seven arrays in Table 3, and the analysis of the errors of the solar azimuth and elevation angles are shown in Figure 8 and Figure 9, respectively. The orientation errors of the arrays that have the same *M* are grouped together with the highlighted orientation error of Array 7, which has the max *M* (M=16). During the clear sky period, the maximum errors of the solar azimuth angle were $5.6^{{}^{\circ}}$, $2.5^{{}^{\circ}}$, and $2^{{}^{\circ}}$ for M=4,8 and 16, respectively (Figure 8); the maximum errors of the solar elevation angle during the clear sky period were $2^{{}^{\circ}}$, $1.2^{{}^{\circ}}$, and $1^{{}^{\circ}}$ for M=4,8, and 16, respectively (Figure 8). It is obvious that the greater the *M*, the smaller the error in solar orientation determination, including errors of the solar azimuth and elevation angles. During the cloudy periods, the errors of all seven arrays, with different *M* in the solar azimuth and elevation angles, are approximately equal, particularly for M=4 and 8, which suggests that the errors of the solar azimuth and elevation angles are approximately independent of *M*. With a clear sky, the interference on each solar panel is primarily generated by internal interference, and with a cloudy sky, the interference is primarily generated by atmospheric scattering. As mentioned in Section 4, the atmospheric scattering can assume many interfering light sources with different directions, and these results confirm that regular pyramid sensor arrays can effectively suppress internal interference by increasing *M* but is invalid for interfering light sources.

## 6. Conclusions

Previous work has found that the accuracy of non-planar sun sensors in solar orientation determination varies with the number of illuminated sensors and the direction of the Sun, but their mathematical relationship is still unknown. They have found methods to suppress zero mean Gaussian noise but methods to eliminate the internal interference and constant interference are not found. We developed a new method to determine the solar orientation in the frequency domain for regular pyramid sun sensors. In addition to proposing two formulations to evaluate the error of the solar elevation angle and the supremum of the error of the solar azimuth angle error in determining the solar orientation, we further found that the error in using regular pyramid sun sensors in determining the solar orientation varies not only with the number of illuminated sensors and the direction of the Sun, but also with the angle between the lateral sides and the bottom of the regular pyramid. The principle of the new method reveals that the errors in solar orientation determination come from the spectrum components of interference at the zero angular frequency and fundamental angular frequencies $\pm \frac{2\pi }{M}$, where *M* is the sensor number of the regular pyramid arrays. In particular, the error of the solar azimuth angle in solar orientation determination only originates from the spectrum component of interference at fundamental angular frequencies. The interference, with bounded power spectra, such as zero-mean Gaussian noise, can be reduced, and even eliminated, by increasing the number of sensors.

Examining the spectrum components of internal interference, we showed that internal interference is inversely proportional to the square root of the sensor number of regular pyramid arrays and can be eliminated by a very large number of sensors. Similarly, for constant interference, such as uniform scattered sunlight in a clear sky, regular pyramid sun sensors will obtain unbiased solar azimuth angle estimation although there is error in the solar orientation determination. Thus, for the internal interference and constant interference, two regular pyramid arrays with orthogonal bottoms can be suggested to achieve an unbiased estimation in determining solar orientation. However, we indicated that external interference is impossible to be completely suppressed by a regular pyramid sun sensors with the spectrum components of external interference. Our field experiment demonstrated that our technique and the traditional methods for determining solar orientation by regular pyramid arrays have the same performance.

## Figures and Tables

**Table 1:** Configurations of positions for the interfering light source.

Position	Azimuth Angle of the Interfering Light Source $a_{e}$ (deg)	Elevation Angle of the Interfering Light Source $r_{e}$ (deg)
1	0	90
2	0.4	45.2
3	21.7	−5

**Table 2:** Configurations of simulated arrays.

Array	Number of Sensor Planes *M*	Angle between the Normal of Sensor Planes and the x-y Plane β (deg)	Azimuth Angle $\alpha_{0}$ of the Normal Vector of Sensor Plane Numbered 0 (deg)
1	16	65	0
2	16	80	0
3	256	65	0
4	256	80	0

**Table 3:** The configurations of seven arrays with their *M*, β and $\alpha_{0}$. Black dots indicate the solar panels being used in each array.

Array	Number of Sensor Planes Mounted on the Lateral Surfaces of the Regular 16-Pyramid																*M*	β (deg)	$\alpha_{0}$ (deg)
	1	2	3	4	5	6	7	8	9	10	11	12	13	14	15	16			
1	•				•				•				•				4	26.4	0
2		•				•				•				•					
3			•				•				•				•				
4				•				•				•				•			
5	•		•		•		•		•		•		•		•		8		
6		•		•		•		•		•		•		•		•			
7	•	•	•	•	•	•	•	•	•	•	•	•	•	•	•	•	16		
